# Invisible contaminants, irreversible consequences? LDPE residues twist the Arabidopsis holobiome

**DOI:** 10.1093/jxb/eraf388

**Published:** 2025-12-04

**Authors:** Swaroop Chakraborty, Zhipeng Guo

**Affiliations:** School of Geography, Earth & Environmental Sciences, University of Birmingham, Edgbaston B15 2TT, UK; College of Life Sciences and Oceanography, Shenzhen University, Shenzhen 518060, China

**Keywords:** Agro-plastics, low-density polyethylene, microplastics, rhizosphere microbiome, transcriptomics

## Abstract

This article comments on:

**Lee D, Lee E, Lee YS, Shin MK, Yang JS, Kim M, Sang MK, Park HJ, Jung HW.** 2025. Agri-plastics in soils drive changes in the rhizosphere bacterial community and plant transcriptome in Arabidopsis. Journal of Experimental Botany **76**, 7003–7025. https://doi.org/10.1093/jxb/eraf336

This article comments on:

**Lee D, Lee E, Lee YS, Shin MK, Yang JS, Kim M, Sang MK, Park HJ, Jung HW.** 2025. Agri-plastics in soils drive changes in the rhizosphere bacterial community and plant transcriptome in Arabidopsis. Journal of Experimental Botany **76**, 7003–7025. https://doi.org/10.1093/jxb/eraf336

**Plastic is an agronomic paradox. Polyethylene mulch film is agriculture’s double-edged sword: doubling tomato yield and halving irrigation water but leaving a legacy of shards and fibres that conventional tillage cannot retrieve. Global surveys report that 0.03–6.7% of small pieces of plastics are piling up in farm soils, sometimes up to 6% of the soil weight, yet crops often show no visible signs of stress under these conditions (Fuller and Gautam, 2016; Stubbins *et al*., 2021; Shokunbi *et al*., 2024; Liang *et al*., 2024). However, recent research by Lee *et al*. (2025) demonstrates that such apparent normalcy is misleading. Their study revealed that when *Arabidopsis thaliana* plants were grown in soils pre-incubated with 5% weather-worn low-density polyethylene (LDPE) for 4 months, they maintained normal growth phenotypes but exhibited significant microbial community shifts in the rhizosphere and transcriptional changes of >2000 key genes, some of which are involved in photosynthesis and nitrogen metabolism. These subtle yet profound alterations in plant physiology and soil ecology may compromise long-term agricultural productivity, underscoring the urgent need for researchers and policymakers to reassess the sustainability of plastic use in modern farming practices.**

## The invisible burden of agricultural plastics

Plastic mulch films, often made of LDPE, are widely used in agriculture to suppress weeds, retain soil moisture, and improve crop productivity. However, their progressive degradation introduces persistent micro- and macroplastic residues into agricultural soils, an insidious form of contamination that escapes visual detection yet exerts measurable impacts at microbial and molecular scales (Briassoulis, 2023). LDPE is generally considered inert, but exposure to UV light, moisture, and microbial action introduces reactive oxygen-containing groups on its surface. These changes enhance the ability of the material to adsorb heavy metals, pesticides, and organic pollutants (Chakraborty *et al.*, 2025; Permana *et al.*, 2025). In the soil, such interactions could turn plastic residues into vectors for co-pollutants, extending their bioavailability and toxicity (Nguyen *et al*., 2023). Plants growing in this altered chemical environment are probably responding not just to the physical presence of plastic but to the complex mix of stressors that they facilitate.

Alarmingly, soil surveys from intensive farming systems in China’s high-input cotton fields document that plastic residues constitute up to 6% of soil dry weight (Liang *et al.*, 2024). Current regulatory paradigms are constrained by conventional pollution metrics, which prioritize visible contamination and acute biomass-based toxicity while neglecting more subtle biological impacts.

The work by Lee *et al*. (2025) challenges this status quo. Through integrated microbiome profiling and transcriptome analysis of Arabidopsis grown in 5% LDPE-contaminated soil, their study uncovers a critical disconnect: while plants maintain normal growth phenotypes, they experience profound dysregulation of root microbial communities and plant gene expression. This paradigm-shifting evidence calls for urgent re-evaluation of how agricultural plastic pollution is defined, monitored, and governed, moving beyond visible indicators to address its hidden biological consequences.

## Subtle changes, profound consequences: uncovering the hidden impacts of LDPE

This study distinguishes itself through its ecologically realistic experimental design. Moving beyond conventional approaches using pristine plastics, Lee *et al*. (2025) implemented a 120 d pre-incubation period for LDPE residues to accurately simulate field weathering processes. This innovative pre-treatment facilitated a natural colonization of plastic surfaces by microbial biofilms, progressive physicochemical changes including surface oxidation and enhanced chemical reactivity, and the formation of environmentally representative microplastic–leachate complexes. The researchers observed a remarkable physiological paradox in Arabidopsis grown in the pre-conditioned soil: (i) apparent normality—no observable changes in plant biomass or morphology; (ii) molecular turmoil—significant differential expression of >2000 genes, particularly those governing photosynthesis, nitrogen assimilation, and oxidative stress response; and (iii) microbial reorganization—16S rRNA sequencing of the rhizosphere revealed compositional shifts favouring bacterial families known for hydrocarbon degradation, such as *Alcanivoracaceae* and *Cytophagaceae*.

These plastic-adapted microbial communities are well documented in marine oil spills (Guo *et al.*, 2024) and compost piles (Salinas *et al*., 2025), but rarely dominate agricultural soils. Their enrichment suggests that aged LDPE fragments create ecological niches for microbes capable of metabolizing hydrocarbons or associated contaminants. The study raises critical questions about the long-term ecological trade-offs. (i) Potential benefit: could plastic-degrading microbes detoxify polymer additives? (ii) Probable cost: might they disrupt plant–microbe mutualisms essential for nutrient acquisition? It remains to be seen whether the enriched microbial consortia exert feedback effects on the host plant, such as modulating hormone signalling, root exudation, or nutrient exchange pathways, which could further destabilize plant–microbe mutualisms under prolonged exposure. (iii) Unanswered questions: what are the cumulative effects on soil health across growing seasons? These findings fundamentally challenge current agricultural plastic management paradigms (i.e. most countries prioritize removing used plastics from fields and replacing petrochemical-based plastics with biodegradable alternatives), demonstrating that standard growth metrics fail to capture complex molecular and microbial disruptions induced by plastic contamination. Beyond plant health, shifts in rhizosphere microbial communities may propagate through the soil food web, affecting nutrient cycling, soil respiration, and even the resilience of agroecosystems to climate stressors.

## Transcriptome–phenotype disconnect: latent stress in plastic-exposed plants

One of the most intriguing aspects of this study is the absence of visible damage of the plants despite deep transcriptional reprogramming. Photosynthesis-related genes such as chlorophyll *a*/*b*-binding protein (light-harvesting systems) and Calvin cycle regulators such as Ribulose small subunit (carbon assimilation) were suppressed, as were nitrate transporters such as *NRT2.5* (nitrogen acquisition). These molecular markers are early indicators of metabolic stress and nutrient limitations.

**Fig. 1.**
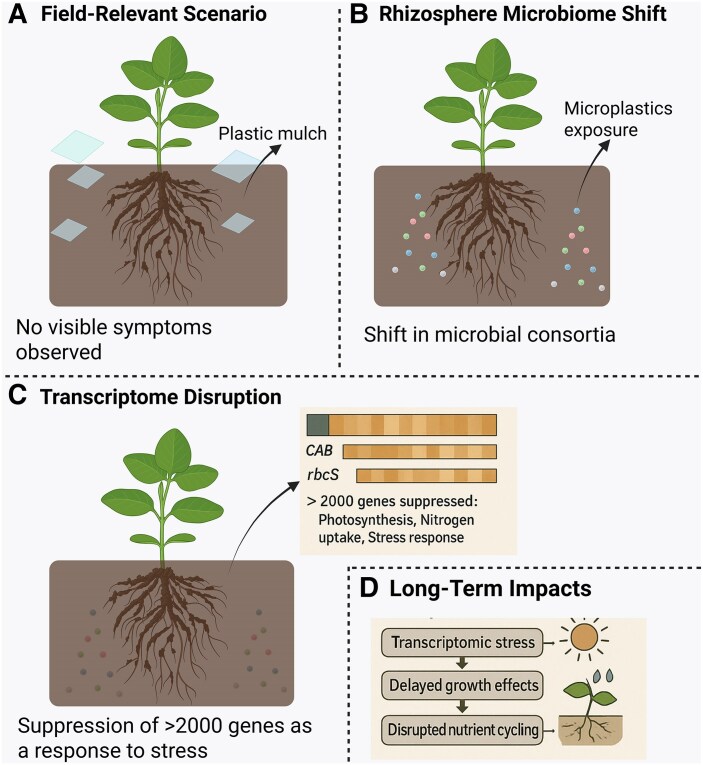
Invisible stress: molecular disruptions beneath the surface. Microplastic contamination from LDPE residues in soil presents a deceptive paradox (A): plants may appear healthy (B), yet their molecular machinery tells another story (C). The study by Lee *et al*. (2025) reveals a striking transcriptome–phenotype disconnect in *Arabidopsis thaliana*. Despite maintaining biomass and morphology, exposed plants exhibited widespread suppression of photosynthesis-related genes [e.g. chlorophyll *a*/*b*-binding protein (*CAB*), small subunit of Rubisco (*rbcS*)] and nutrient transporter genes (*NRT2.5*), alongside shifts in the rhizosphere microbial community toward plastic-degrading taxa (C). These findings suggest that traditional indicators such as yield or chlorophyll content may be insufficient to detect early-stage plastic-induced stress (D). Omics-based biomarkers offer a powerful lens to detect latent dysfunction before visible damage occurs and may serve as essential tools for sustainable agroecosystem management in the age of microplastic pollution. The Figure was prepared using Biorender software.

The lack of obvious phenotypic symptoms could be explained by compensatory mechanisms within the plant, whereby internal reserves of carbon and nitrogen are buffers against short-term disruptions (Zhao *et al*., 2023). However, longitudinal studies in lettuce and maize have shown that similar transcriptomic changes eventually translate into stunted growth, reduced yields, and altered nutrient profiles when exposure is prolonged (Graf *et al*., 2023; Wang *et al*., 2023). Thus, the findings by Lee *et al*. (2025) may represent the beginning of a cascade that, under field conditions, would culminate in agronomic losses. While the study presents robust transcriptomic and microbial profiling, functional assays such as metabolomic validation or nutrient uptake measurements would be valuable to confirm whether gene expression changes translate into physiological impairment. This decoupling of gene expression changes and non-obvious phenotypical symptoms underscores the need to rethink how we define ‘plant health’ in the context of chronic stressors, advocating omics-informed metrics that can detect latent dysfunction. Despite the absence of visible damage, transcriptional reprogramming affected core pathways such as photosynthesis and nitrogen uptake (Fig. 1), where apparent plant health masks deep molecular dysfunction.

## Field relevance and limitations

Although the Arabidopsis model is powerful for dissecting genetic responses, its shallow root system and non-mycorrhizal status limit direct extrapolation to field-grown crops. Nevertheless, the study aligns with observations from larger mesocosm experiments showing that plastic residues at 0.5–2% levels can alter rhizosphere microbial composition and reduce nitrogen fixation in crops such as wheat and soybean (Qi *et al*., 2020; Porto *et al*., 2024). Distinguishing the role of plastic-derived leachates versus the physical presence of polymer particles would further refine our understanding of the primary stressors, an aspect that future studies should explicitly address through appropriate abiotic controls. Furthermore, the choice of a 5% plastic loading is justified by real-world contamination levels in certain high-input systems, even if it exceeds the average for most temperate fields. Importantly, Lee *et al*. (2025) acknowledge this and frame their findings as a conservative estimate of risk, one that could be exacerbated under conditions of drought, fertilizer overuse, or high temperatures.

## Towards plastic-smart agriculture

What should be done? First, we must expand our understanding of plastic toxicity beyond classical ecotoxicological endpoints. Molecular biomarkers such as transcriptomic shifts or changes in microbial functional guilds offer early-warning systems that can detect stress before yield losses occur. The findings arrive at a pivotal moment, as organizations such as the Food and Agriculture Organization of the United Nations (FAO) and the United Nations Environment Programme (UNEP) are calling for stricter regulatory frameworks to manage agroplastic residues, especially considering emerging evidence linking microplastic pollution to disrupted plant–soil feedback loops. Second, there is a pressing need for standardized testing frameworks that incorporate realistic ageing, soil types, and plant species. Many biodegradable alternatives to LDPE such as polybutylene adipate terephthalate (PBAT) or polylactic acid (PLA) blends are being marketed as sustainable solutions, yet studies have shown they can also disrupt microbial networks and persist under anaerobic or alkaline conditions (Xu *et al*., 2024). Any replacement material must therefore be evaluated using comprehensive, multi-omics approaches. Moreover, purportedly biodegradable plastics such as PBAT and PLA blends may persist under common agricultural conditions, and their additives or degradation intermediates have shown potential for microbial toxicity, warranting equally rigorous scrutiny. Third, mitigation strategies must combine upstream design and downstream management. Policies should encourage film retrieval before fragmentation, promote soil health monitoring, and support farmers in adopting more sustainable mulching practices. Incentives for recycling and extended producer responsibility schemes could also help close the loop on plastic use. Further, implementing robust monitoring and management systems could provide a more sustainable strategy for safeguarding agroecosystem health. Finally, we must recognize that plastic pollution is not a standalone issue. It intersects with climate change, food security, and biodiversity loss. As global temperatures rise and weather patterns become more erratic, the synergistic impacts of heat stress and plastic contamination may further destabilize cropping systems. Developing resilient agroecosystems will require integrating material science, soil microbiology, plant physiology, and socio-economic planning.

## A silent legacy uncovered

Lee *et al*. (2025) have peeled back the soil surface to reveal a hidden dimension of plastic pollution. Their work shows that even in the absence of visible harm, plastic residues can undermine the very foundations of plant health by reshaping microbial communities and reprogramming gene expression. These findings challenge the assumption that what we cannot see does not matter, and they call for a recalibration of how we manage plastics in agriculture. Moving forward, research must prioritize systems-level approaches that capture the complexity of plant–soil–plastic interactions. Policymakers must act swiftly to translate emerging science into practical regulation. As scientists we must continue to illuminate the invisible threads that tie materials to microbes, genes to yields, and residues to resilience. The silent legacy of LDPE in our soils is a call to action, not just to reduce pollution, but to reimagine a farming future where productivity and planetary health go hand in hand.
